# Sexual function outcomes in prostate and cervical cancer patients treated with radiotherapy in sub-Saharan Africa: A cross-sectional study

**DOI:** 10.1371/journal.pone.0324662

**Published:** 2025-05-20

**Authors:** Joseph Daniels, Kofi Adesi Kyei, Prince Dela Frimpong-Boateng, Andrew Yaw Nyantakyi

**Affiliations:** 1 National Centre for Radiotherapy, Oncology and Nuclear Medicine, Korle-Bu Teaching Hospital, Accra, Ghana; 2 Department of Radiography, University of Ghana, Legon, Accra, Ghana; Tarbiat Modares University, IRAN, ISLAMIC REPUBLIC OF

## Abstract

Radiotherapy is indispensable for the successful treatment of many pelvic malignancies, but it is often associated with significant adverse effects on sexual function, including vaginal stenosis, decreased lubrication, erectile dysfunction, and loss of libido. Sexuality and sexual function are important aspects of quality of life for cancer survivors, yet sexual dysfunction remains an underreported complication among patients, particularly those treated for prostate and cervical cancers in limited-resource settings. This quantitative cross-sectional study aimed to evaluate sexual function outcomes among 144 prostate and 160 cervical cancer patients treated with pelvic radiotherapy at a major cancer treatment centre in sub-Saharan Africa. Data were collected using questionnaires based on the Female Sexual Function Index and the International Index of Erectile Function. Data were coded, cleaned, and analyzed using STATA statistical software package (version 17). The mean age of the patients with cervical cancer was 53.5 years (SD 9.6) ranging from 37 to 69 years whereas the mean age of patients with prostate cancer was 67.1 years (SD 7.7) ranging from 56 to 79 years. Among female participants, 79.9%, had moderate to severe sexual dissatisfaction, 94.4% had poor or no satisfactory orgasm whereas 97.2% had difficulty with lubrication. Only 5.6%, 5.6%, 2.8%, and 20.1% of the female participants achieved sexual desire, orgasm, lubrication, and overall satisfaction respectively. In all, 94.4% of the female participants had a total FSFI score less than 26 whereas 5.6% had a score greater than 26. Most patients did not indulge in sexual activity. There was a high prevalence of sexual dysfunction across multiple domains, with cervical cancer patients experiencing diminished desire, poor arousal, lubrication difficulties, and impaired orgasmic function, leading to low relationship satisfaction and avoidance of sexual activity. Similarly, prostate cancer patients demonstrated severe erectile dysfunction, low sexual desire, and dissatisfaction with intercourse and overall sexual experience.

## Introduction

Cancer is one of the leading causes of morbidity and mortality worldwide, significantly impacting patients’ quality of life [1]. Prostate cancer, the most common cancer among men, and cervical cancer, a leading cause of cancer-related deaths among women in limited-resource regions, are both highly prevalent in Ghana [2]. Prostate cancer accounted for 13.4% of all new cancer cases among men in Ghana in 2022 alone, whereas cervical cancer accounted for 32.5% of new cancer cases among women, highlighting the critical need for effective preventive, screening and management strategies [3].

Sexual health, influenced by biological, psychological, and social factors, is a crucial component of overall well-being, with a growing body of evidence showing that cancer can dramatically affect the sexuality, sexual functioning, relationships, and the “sense of self” of patients [4]. Sexual dysfunction is a prevalent but often underreported complication among cancer survivors, particularly those treated with curative intent for prostate and cervical cancers (pelvic malignancies).

Radiotherapy, while effective in targeting cancerous cells, can inadvertently affect adjacent healthy tissues and organs, potentially leading to a wide range of side effects. One of the less discussed but highly impactful side effects is its potential to impair sexual function [5,6]. For prostate cancer patients, radiation therapy can lead to erectile dysfunction, decreased libido, loss of penile length, and other sexual health issues [5,7,8]. The standard curative treatment for locally advanced cervical cancer is concurrent chemo-radiotherapy followed by brachytherapy which may lead to vaginal dryness, stenosis, and dyspareunia. Treatment-related sexual dysfunction can lead to emotional distress, relationship challenges, and reduced quality of life. Beyond the physiological effects, psychological distress, anxiety, depression, and relationship strain further exacerbate sexual dysfunction.

In limited-resource healthcare settings, the stigma associated with discussing sexual health issues further complicates the management of these side effects, often leaving patients without the necessary support and resources to address their concerns. While advancements in medical interventions have improved survival rates for prostate and cervical cancers, the long-term side effects, particularly on sexual function, remain a significant concern [6]. The aim of the study was to evaluate sexual function outcomes among prostate and cervical cancer patients treated with radiotherapy in a limited-resource healthcare setting.

## Methods

### Study design and setting

The research was a descriptive, quantitative, cross-sectional study conducted at a leading cancer treatment centre in sub-Saharan Africa. The high treatment capacity of the study site offered access to a substantial number of prostate and cervical cancer patients undergoing radiotherapy, ensuring adequate sample size and study feasibility. Additionally, the radiotherapy centre’s multidisciplinary cancer care framework facilitated comprehensive patient assessment, making it a suitable setting for evaluating the impact of pelvic radiotherapy on sexual function.

### Participants

The study population comprised adult female patients with a histologically confirmed diagnosis of cervical cancer and male patients with prostate cancer who were treated with radiotherapy between January 01 and December 31, 2023. The study included only eligible patients with verified histopathology reports and confirmed absence of metastasis. For patients with cervical cancer, distant metastatic status was evaluated with computed tomography imagining of the chest and abdomen whereas patients with prostate cancer were evaluated with either a bone scan, whole body magnetic resonance imaging or single photon emission computed tomography. Patients treated with palliative intent were excluded from the study, likewise those with pre-existing sexual dysfunction, previous history of pelvic surgery or comorbidities affecting sexual function.

### Sampling method

Stratified purposive sampling was employed to ensure adequate representation of both prostate and cervical cancer patients undergoing radiotherapy. This approach enhanced the depth and relevance of the data collected. First, the study population was divided into two distinct strata based on cancer type: prostate and cervical cancer. This stratification ensured that both groups who may have different treatment experiences and outcomes, were adequately represented in the study. Within each stratum, participants were purposefully selected based on predefined inclusion and exclusion criteria to enhance the study’s validity and representativeness. The purposive component allowed the selection of individuals who could provide rich, relevant, and diverse insights into the research questions, particularly around survival outcomes, quality of life, and treatment-related side effects.

### Study size

The sample size was determined using the Taro Yamane formula: n = *N*/ (1 + *N*(e^2^)), where “*N”* was the estimated population size, and “e” was the margin of error (5%). Based on this calculation, 142 cervical and 158 prostate cancer patients were required. In all, 320 questionnaires were administered to 170 prostate and 150 cervical cancer patients at an overall response rate of 95% (160 males and 144 females).

### Data collection

Patients self-administered validated and standardized questionnaires to assess their sexual function. “The Female Sexual Function Index (FSFI) consisting of 16 items comprising six sub-scales (desire, arousal, lubrication, orgasm, satisfaction, pain) [9] was used to evaluate sexual function in patients with cervical cancer. This questionnaire is a validated research instrument for assessing clinically diagnosed female sexual dysfunction and has been demonstrated to discriminate reliably between women with and without female sexual arousal disorder in each of the five domains [10]. The FSFI tool was assessed for reliability in the study setting using Cronbach’s alpha, yielding a value of 0.89, within the acceptable range (≥ 0.85). Additionally, test-retest reliability was examined over a two-week period, demonstrating strong reproducibility of responses within the study population. The multi-dimensional International Index of Erectile Function (IIEF) questionnaire was used to evaluate sexual function in patients with prostate cancer. The IIEF questionnaire has sub-scales of erectile function, orgasmic function, sexual desire, intercourse satisfaction, and overall satisfaction. This research instrument has also been validated in all five domains with a high degree of sensitivity and specificity to the effects of treatment [9,11] on male sexual function. The reliability of the IIEF was also evaluated using Cronbach’s alpha, which demonstrated a high internal consistency across its domains. A Cronbach’s alpha value of 0.90 was obtained, confirming its robustness in assessing erectile function, reinforcing its reliability in the population.

### Statistical Analysis

Data were coded, cleaned, and analysed using STATA statistical software package version 17 for Microsoft Windows (College Station, TX: StataCorp LLC). Descriptive statistics, including mean and standard deviation, were used to summarize continuous variables, while categorical variables were presented as frequencies and percentages. Normality of continuous variables was assessed using the Shapiro-Wilk test. The grand mean was determined for each domain by calculating the weighted average of the mean scores across subgroups. This involved summing the mean scores of individual subgroups, each multiplied by its respective sample size, and dividing by the total sample size. The grand mean provided an overall measure of central tendency across the study population, facilitating comparisons between different domains. Higher grand mean values indicated better overall function or outcomes within a given domain, while lower values suggested greater impairment.

### Bias

The study acknowledges several potential sources of bias that may influence the findings. Recall bias is of particular concern, as participants self-reported their sexual function, which could lead to underreporting or overreporting due to social desirability or memory limitations. Measurement bias could also exist, as the FSFI and IIEF were developed in Western populations and may not have fully captured the cultural and contextual nuances of sexual health in sub-Saharan Africa. Additionally, cultural bias may have influenced responses, as discussing sexual dysfunction remains stigmatized in Ghana, potentially leading to reluctance in providing accurate information. Self-administering the questionnaires, minimized cultural bias by reducing the influence of the researcher’s presence. This approach also allowed participants to interpret and respond to questions in a way that aligned with their personal experiences without feeling pressured to conform to perceived societal norms. Participation bias was addressed by recruiting only eligible patients who voluntarily provided written informed consent. The use of validated tools also mitigated bias, ensuring the consistency and reliability of the findings.

### Ethical considerations

Ethical approval was obtained from the Institutional Review Board of the School of Biomedical and Allied Health Sciences, University of Ghana (SBAHS/AA/RAD/10681693/2023). All patients involved in the study provided written informed consent. Data were anonymized before analysis, ensuring confidentiality and compliance with ethical guidelines.

## Results

### Participants

**Fig 1:**
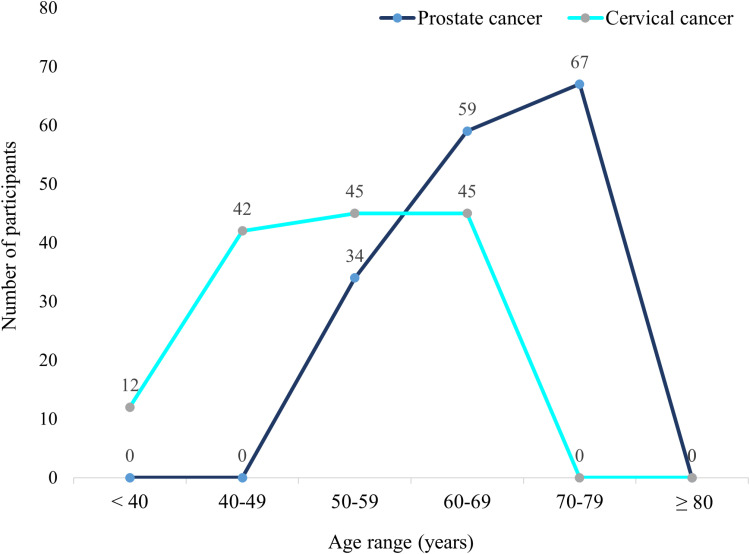
Age distribution of prostate and cervical cancer patients.

The mean age of participants with cervical cancer was 53.5 years (SD 9.6) ranging from 37 to 69 years whereas the mean age of patients with prostate cancer was 67.1 years (SD 7.7) ranging from 56 to 79 years. Among participants with prostate cancer, 21.2% were between 50–59 years, whereas 41.9% were between 70–79 years indicating a higher prevalence in older men. In contrast, among patients with cervical cancer, 8.3% were younger than 40 years, 29.2% were between 40–49, indicating a higher prevalence in relatively younger women (Fig 1).

### Sexual function of (female) patients with cervical cancer

Table 1 summarizes the FSFI scores of patients with cervical cancer. In the desire domain, 86.1% of the participants reported “almost never or never” experiencing sexual desire, indicating a significant lack of libido (mean = 1.25, SD 0.76). In all, 94.4% reporting “no sexual activity”. Confidence and satisfaction with arousal were both low, with mean scores of 1.5 (SD 0.5) each. Difficulties with lubrication were common, as among those who were sexually active (5.6%), only 2.8% reported lubrication “most times” or “a few times”, suggesting suboptimal physical readiness for intercourse. The mean lubrication score was 3.0 (SD 1.0). Similarly, orgasm function was compromised, 5.6% of those who attempted sexual activity reporting difficulty reaching orgasm (mean = 3.00, SD 0). Among those engaging in intercourse, satisfaction with emotional closeness scored a mean of 2.50 (SD 0.5), whilst satisfaction with sexual relationships was generally low, with 59.8% reporting moderate to severe dissatisfaction (mean = 2.38, SD 1.26). Discomfort or pain was minimal, but this was largely due to the lack of sexual activity (mean = 1.50, SD = 0.50).

**Table 1:** The Female Sexual Function Index scores of patients with cervical cancer (N = 144).

Domain	Score	n	%	Mean (X̄)	SD
**Desire**					
**Frequency of sexual desire**					
Almost always or always	5	4	2.8	1.25	0.76
Most times	4	0	–		
Sometimes	3	4	2.8		
A few times	2	12	8.3		
Almost never or never	1	124	86.1		
**Arousal**					
**Frequency of arousal**					
No sexual activity	0	136	94.4	2.0	0
Almost always or always	5	0	–		
Most times	4	0	–		
Sometimes	3	0	–		
A few times	2	8	5.6		
Almost never or never	1	0	–		
**Confidence about arousal**					
No sexual activity	0	136	94.4	1.5	0.5
Very high confidence	5	0	–		
High confidence	4	0	–		
Moderate confidence	3	0	–		
Low confidence	2	4	2.8		
Very low or no confidence	1	4	2.8		
**Satisfaction with arousal**					
No sexual activity	0	136	94.4	1.5	0.5
Almost always or always	5	0	–		
Most times	4	0	–		
Sometimes	3	0	–		
A few times	2	4	2.8		
Almost never or never	1	4	2.8		
**Lubrication**					
**Lubrication during sex**					
No sexual activity	0	136	94.4	3.0	1.0
Almost always or always	5	0	–		
Most times	4	4	2.8		
Sometimes	3	0	–		
A few times	2	4	2.8		
Almost never or never	1	0	–		
**Difficulty with lubrication**					
No sexual activity	0	136	94.4	3.0	1.0
Extremely difficult/impossible	1	0	–		
Very difficult	2	4	2.8		
Difficult	3	0	–		
Slightly difficult	4	4	2.8		
Not difficult	5	0	–		
**Maintenance of lubrication**					
No sexual activity	0	136	94.4	3.0	1.0
Almost always or always	5	0	–		
Most times	4	4	2.8		
Sometimes	3	0	–		
A few times	2	4	2.8		
Almost never or never	1	0	–		
**Difficulty maintaining lubrication**					
No sexual activity	0	136	94.4	3.0	1.0
Extremely difficult/impossible	1	0	–		
Very difficult	2	4	2.8		
Difficult	3	0	–		
Slightly difficult	4	4	2.8		
Not difficult	5	0	–		
**Orgasm**					
**Frequency of orgasm**					
No sexual activity	0	136	94.4	3.0	0
Almost always or always	5	0	–		
Most times	4	0	–		
Sometimes	3	8	5.6		
A few times	2	0	–		
Almost never or never	1	0	0		
**Difficulty reaching orgasm**					
No sexual activity	0	136	94.4	3.0	0
Extremely difficult/impossible	1	0	–		
Very difficult	2	0	–		
Difficult	3	8	5.6		
Slightly difficult	4	0	–		
Not difficult	5	0	–		
**Satisfaction with orgasm**					
No sexual activity	0	136	94.4	3.0	0
Very satisfied	5	0	–		
Moderately satisfied	4	0	–		
Neutral	3	8	5.6		
Moderately dissatisfied	2	0	–		
Very dissatisfied	1	0	–		
**Satisfaction**					
**Satisfaction with emotional closeness**					
No sexual activity	0	136	94.4	2.5	0.5
Very satisfied	5	0	–		
Moderately satisfied	4	0	–		
Neutral	3	4	2.8		
Moderately dissatisfied	2	4	2.8		
Very dissatisfied	1	0	–		
**Satisfaction with relationship**					
Very satisfied	5	12	8.3	2.38	1.26
Moderately satisfied	4	17	11.8		
Equally satisfied and dissatisfied	3	29	20.1		
Moderately dissatisfied	2	41	28.5		
Very dissatisfied	1	45	31.3		
**Overall satisfaction**					
Very satisfied	5	12	8.3	2.38	1.26
Moderately satisfied	4	17	11.8		
Equally satisfied and dissatisfied	3	29	20.1		
Moderately dissatisfied	2	41	28.5		
Very dissatisfied	1	45	31.3		
**Discomfort/Pain**					
**Discomfort/Pain**					
Did not attempt intercourse	0	136	94.4	1.5	0.5
Almost never or never	1	4	2.8		
A few times	2	4	2.8		
Sometimes	3	0	0		
Most times	4	0	0		
Almost always or always	5	0	0		

**Fig 2:**
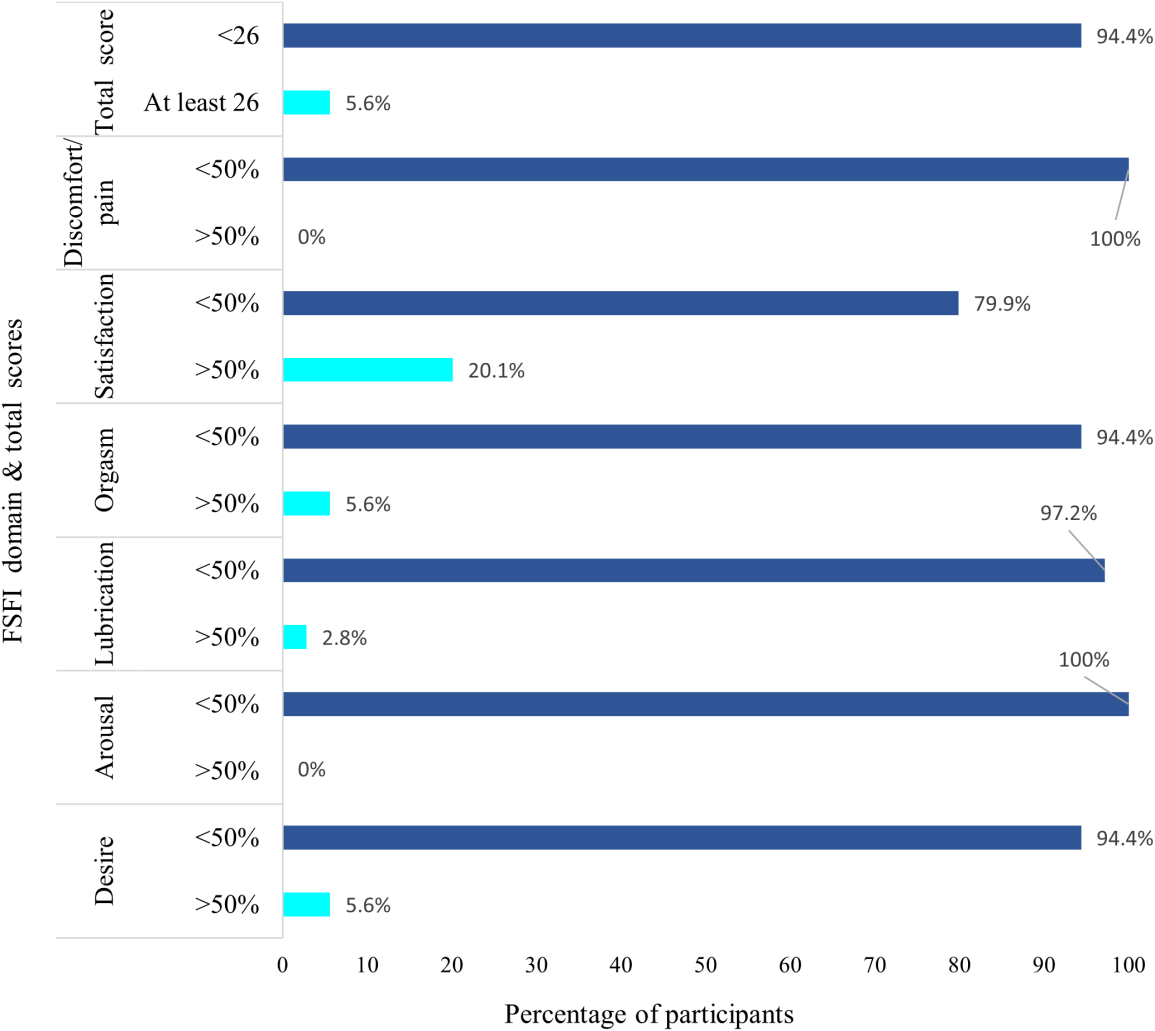
Distribution of female sexual function index (FSFI) scores of participants with cervical cancer across various domains.

Fig 2 illustrates the percentage distribution of participants across different domains of the female sexual function index, highlighting key areas of sexual health. For the total score, 94.4% of participants scored below 26, indicating potential dysfunction, with only 5.6% scoring at least 26. In the desire domain, 94.4% had low scores, while only 5.6% scored above 50%, suggesting limited desire. Arousal showed no participants scoring above 50%, with all participants at or below the 50% threshold. Lubrication was similarly low, with 97.2% below 50%, and only 2.8% above. Orgasm results revealed 94.4% of participants scored below 50%, with 5.6% achieving higher scores. In terms of satisfaction, 20.1% reported higher satisfaction levels whereas 79.9% scored below 50%. Lastly, discomfort/pain was low across participants, with 100% scoring below the 50% mark, indicating minimal reported discomfort or pain.

### Sexual function of (male) patients with prostate cancer

Male sexual function was assessed using a 5-point Likert scale ranging from 1–5 with higher scores indicating greater level of sexual functioning on the respective domain. Table 2 provides a comprehensive overview of the IIEF scores for 160 male participants diagnosed with prostate cancer, across five domains: confidence in achieving erectile function, orgasmic function, sexual desire, satisfaction with intercourse, and overall satisfaction. The overall weighted IIEF score was 4.75, reflecting substantial impairments in multiple aspects of sexual function. Confidence in achieving an erection was generally low, with a mean score of 2.07 (SD 1.19). A majority of participants (73.2%) reported very low to low confidence, while only 10.6% expressed moderate or higher confidence in achieving erections. Orgasmic function was also severely impaired (mean = 1.37, SD 0.74), with 78.8% reporting they almost never or never experienced orgasm. Similarly, sexual desire was notably diminished (mean = 1.95, SD 0.89), as 73.8% of participants reported very low or low desire, and only 5.6% had high or very high levels. Satisfaction with intercourse was particularly poor, with a mean score of 1.37 (SD 0.74). Nearly 79% of participants reported no enjoyment, and only 15.6% found it “fairly enjoyable,” with no reports of high or very high satisfaction. Overall sexual satisfaction (mean = 1.99, SD 1.08) was similarly low, with 47.5% of participants being very dissatisfied and only 10.6% reporting moderate satisfaction.

**Table 2:** The International Index of Erectile Function scores of male participants (N = 160).

Domains	Score	n	%	Mean (SD)
**Confidence to get erectile function**				
Very low	1	67	41.9	2.07 (1.21)
Low	2	50	31.3	
Moderate	3	17	10.6	
High	4	17	10.6	
Very High	5	9	5.6	
**Orgasmic function**				
Almost never or never	1	126	78.8	1.38 (0.75)
A few times	2	8	5.0	
Sometimes	3	26	16.2	
Most times	4	0	0	
Almost always or always	5	0	0	
**Sexual desire**				
Very low	1	59	36.9	1.95 (0.90)
Low	2	59	36.9	
Moderate	3	33	20.6	
High	4	9	5.6	
Very High	5	0	0	
**Satisfaction with intercourse**				
No enjoyment	1	126	78.8	1.37 (0.74)
Not very enjoyable	2	9	5.6	
Fairly enjoyable	3	25	15.6	
Highly enjoyable	4	0	0	
Very highly enjoyable	5	0	0	
**Overall satisfaction**				
Very dissatisfied	1	76	47.5	2.0 (1.08)
Moderately dissatisfied	2	25	15.6	
About equally satisfied and unsatisfied	3	42	26.3	
Moderately Satisfied	4	17	10.6	
Very satisfied	5	0	0	

**Fig 3:**
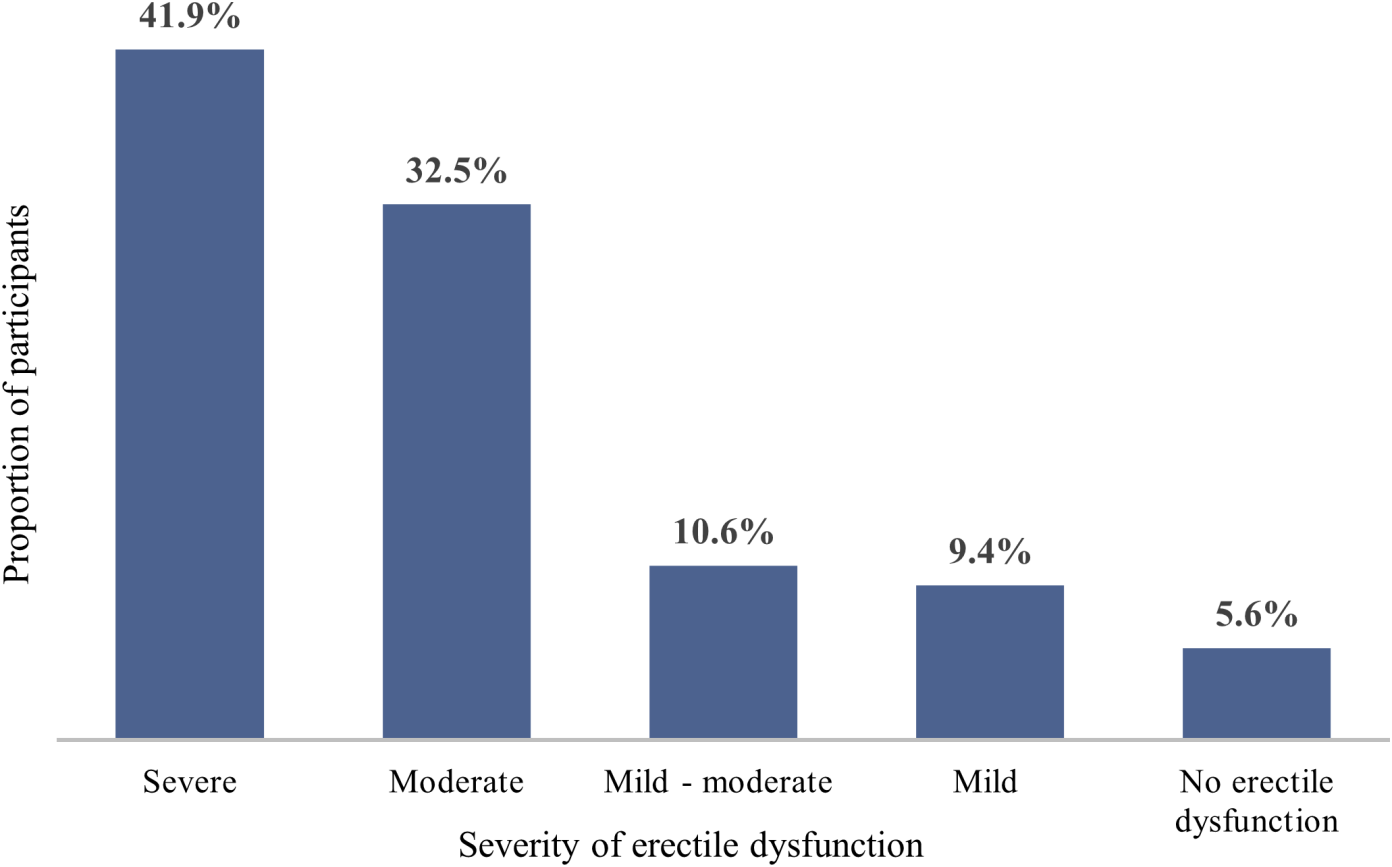
Severity of erectile dysfunction among participants with prostate cancer.

Fig 3 illustrates the proportion of patients with prostate cancer with various severities of erectile dysfunction. The largest group, 41.9%, experienced severe erectile dysfunction (scores between 1 and 7), followed by 32.5% with moderate erectile dysfunction, scoring between 8 and 11. A small portion, 5.6%, reported no erectile dysfunction (scores between 22 and 25). This distribution indicates that most participants suffered from significant erectile dysfunction, with severe and moderate levels being most common.

## Discussion

The study revealed significant sexual function impairments among patients with prostate and cervical cancer treated with radiotherapy, impacting multiple domains and overall quality of life. Cervical cancer patients, who were relatively younger (mean age: 53.5 years, SD 7.7) faced widespread sexual dysfunction, with 94.4% reporting low sexual desire (mean = 1.25, SD 0.76), lubrication difficulties (mean = 3.0, SD 1.0), and impaired orgasmic function (mean = 3.0, SD 0). Relationship satisfaction was low (mean = 2.38, SD 1.26). While discomfort or pain was minimal (mean = 1.5, SD 0.5), this was largely due to a lack of sexual activity, as 94.4% had no sexual engagement. Prostate cancer patients, predominantly older men (mean age: 67.1 years, SD 9.6), exhibited low confidence in achieving erections (mean = 2.07, SD 1.19), poor orgasmic function (mean = 1.37, SD 0.74), diminished sexual desire (mean = 1.95, SD 0.89), high dissatisfaction with intercourse (mean = 1.37, SD 0.74) and overall sexual experience (mean = 1.99, SD 1.08). Severe to moderate erectile dysfunction was prevalent, with 73.2% reporting low confidence in achieving erections, 79% experiencing orgasmic dysfunction, and nearly 50% expressing dissatisfaction with their sexual experiences.

The findings highlight distinct demographic patterns for prostate and cervical cancer patients treated with radiotherapy in sub-Saharan Africa, reflecting the age-related disparities between the two cancers. Prostate cancer was predominantly seen in older men, which aligns with global trends indicating that prostate cancer incidence increases significantly with age, likely due to cumulative genetic mutations and hormonal changes in older men [12]. Conversely, cervical cancer was more prevalent in relatively younger women. This pattern is consistent with the earlier onset of cervical cancer, influenced by risk factors such as human papillomavirus infection, which is common in women of reproductive age [13]. These age differences are significant when considering sexual function outcomes post-radiotherapy. Older prostate cancer patients might already experience age-related declines in sexual function, compounding the impact of treatment-induced dysfunction [14].

Overall, there were severe impairments across multiple domains of sexual function in female cervical cancer patients post-radiotherapy, highlighting critical challenges among patients treated for cervical cancer, pertaining to desire, arousal, lubrication, orgasm, satisfaction, and discomfort/pain. Nearly all participants (94.4%) reported a lack of sexual activity, with 86.1% experiencing a profound decline in sexual desire. Cervical cancer and its treatment modalities, such as radiotherapy, can result in hormonal changes, contributing to diminished libido [15]. Treatment-induced hormonal changes, including estrogen depletion, alongside psychological distress such as fear of recurrence and body image concerns, may also contribute to diminished libido [16]. Furthermore, societal, and cultural factors, including stigma associated with cervical cancer and its treatment, may possibly exacerbate the loss of sexual interest.

Arousal and lubrication difficulties were widespread, with only 2.8% reporting any level of confidence or satisfaction regarding arousal. These issues may be attributed to radiation-induced damage to the pelvic structures, including neurovascular pathways essential for sexual response [17]. Lubrication impairment was also common, likely due to radiation-induced vaginal atrophy, dryness, and stenosis, making sexual activity difficult and often painful [18]. Orgasmic dysfunction was equally prevalent (5.6% found it difficult to achieve orgasm), with physical factors such as loss of sensation due to nerve damage and psychological factors, including anxiety and depression playing significant roles [19,20]. These complications can result in discomfort during sexual activity, further reducing the likelihood of engaging in intimate relationships [21]. The discomfort/pain domain revealed that 94.4% of participants did not attempt intercourse, with only 2.8% reporting pain “almost never.” While pain was not a major concern for most participants, the lack of sexual activity highlights a broader issue: many women avoid intercourse entirely due to anticipated or experienced pain, compounded by emotional distress and physical changes post-treatment.

Relationship dissatisfaction was another major concern, with 28.5% of patients moderately dissatisfied and 31.3% experiencing very high dissatisfaction. Lack of sexual activity, combined with emotional distress, alters partner dynamics, and societal stigma, often resulting in strained relationships and social withdrawal [22]. Comparative studies from Nigeria [18] and Senegal [19] have reported similar trends, with up to 91% of cervical cancer survivors experiencing diminished sexual satisfaction, often leading to marital difficulties. In contrast, Western studies report lower rates of complete sexual inactivity; for example, Müller et al. (2021) found that while 80% of European cervical cancer patients had some form of sexual dysfunction, only 60% completely ceased sexual activity [23]. This suggests that sociocultural factors in sub-Saharan Africa may amplify post-treatment sexual challenges.

These findings underscore the need for a multidisciplinary approach to address sexual health challenges in female cervical cancer patients. Patients and their partners can benefit from education about the normalcy of these changes and potential coping mechanisms. Including partners in counselling sessions can foster open communication and improve relational satisfaction. Strengthening intimacy and emotional closeness can also enhance overall sexual health outcomes significantly. Physiotherapy, vaginal dilators, and lubricants should be offered to manage the physical sequelae of radiotherapy, such as dryness, stenosis, and loss of elasticity.

The study highlights a significant impact of radiotherapy on sexual function in prostate cancer patients, with severe to moderate erectile dysfunction affecting 74.4% of the participants. This aligns with prior research emphasizing that radiotherapy often results in vascular and neurogenic damage leading to erectile dysfunction [24,25]. Confidence in achieving erections was strikingly low, with 73.2% reporting low confidence, and nearly half of the participants expressing dissatisfaction with their overall sexual experience. The results align with prior studies from sub-Saharan Africa, including a Nigerian study [26], which reported a 70% prevalence of moderate to severe erectile dysfunction post-radiotherapy, and another study in South Africa [27], which demonstrated that 75% of prostate cancer survivors had diminished sexual desire and difficulties with orgasmic function. Comparative studies from Ghana and Côte d’Ivoire further reinforce these results. Addai et al. (2021) reported that 72% of prostate cancer patients undergoing radiotherapy experienced erectile dysfunction, often accompanied by psychological distress and reduced self-esteem[28]. Kouadio et al. (2022) observed similar trends, where cultural stigma discouraged many patients from seeking medical assistance [29]. In Western settings, while erectile dysfunction remains prevalent, access to medical interventions such as penile rehabilitation, counseling, and pharmacological treatments mitigates its severity. For instance, Smith et al. (2021) in the United States found that while 65% of prostate cancer survivors experienced erectile dysfunction, nearly half reported improvement with available medical and psychological interventions—options that are often less accessible in sub-Saharan Africa [30].

Nearly 79% of participants experienced orgasm “almost never” or “never”, with no participants reporting consistent orgasmic function. These findings are consistent with studies that highlight the adverse effects of radiotherapy on orgasmic function, likely due to nerve damage and psychological stress associated with cancer treatment [31]. There were low levels of sexual desire, with 36.9% reporting very low and an equal proportion reporting low desire. Only 20.6% experienced moderate levels, and none reported high or very high sexual desire. This is reflective of the interplay between hormonal changes induced by cancer treatment and psychological distress, which are known to reduce libido in men undergoing radiotherapy [32]. Satisfaction with intercourse was markedly low, with nearly 79% reporting no enjoyment. Overall sexual satisfaction mirrored these results, with nearly half of the participants expressing being very dissatisfied. These findings suggest that the physical limitations imposed by treatment, combined with emotional distress, contribute to the high levels of dissatisfaction observed [33]. The high prevalence of erectile dysfunction underscores the critical need for integrative care models that address both physical and psychosocial aspects of sexual health in prostate cancer patients. Counseling, sexual rehabilitation programs, and treatment options like phosphodiesterase inhibitors, penile prostheses, or vacuum devices should be considered to improve sexual outcomes and overall quality of life.

### Limitations

The study was conducted in a single healthcare institution, which may not represent the experiences of cervical and prostate cancer patients in other regions or healthcare systems. The non-population-based design limits the generalizability of the findings. Secondly, the relatively small sample size limits the statistical power to detect nuanced differences in sexual function domains. The reliance on self-reported questionnaires may have introduced recall and reporting biases. Also, variables such as age, treatment duration, and disease duration, each of which could influence sexual function, were not controlled for in the analysis.

The study also relied on self-reported data collected through standardized questionnaires (IIEF and FSFI), which may be subject to recall bias and social desirability bias. While the FSFI comprehensively assesses multiple dimensions of female sexual function, the IIEF, although widely validated, primarily focuses on erectile function, orgasmic function, sexual desire, intercourse satisfaction, and overall satisfaction. It does not encompass other important aspects of male sexual health, such as ejaculatory function, pain during intercourse, or psychological distress related to sexual dysfunction. A more comprehensive evaluation of male sexual function may require additional assessment tools beyond the IIEF. However, given the absence of culturally specific alternatives, the use of the IIEF and FSFI tools in the cultural setting of the study population is both justifiable and necessary to facilitate clinically meaningful assessments of sexual function. The study demonstrates their applicability in the West African population and underscore the need for future validation studies in this region.

The cross-sectional nature of the study precludes establishing causality between treatment with radiation and sexual dysfunction. Longitudinal studies would be better suited to explore changes over time and the potential impact of interventions. Moreover, the absence of a control group, such as cancer survivors not undergoing radiotherapy or individuals without cancer limits the ability to isolate the specific effects of the disease and its treatment on sexual function. Sexual dysfunction in cancer patients is multifaceted, influenced by biological (hormonal changes, nerve damage), psychological (depression, anxiety), and social factors (relationship dynamics, cultural stigma). The inclusion of bio-psycho-social assessments in future research is necessary to fully understand and address these challenges.

## Conclusions

The study showed the occurrence of significant sexual function impairments among prostate and cervical cancer patients post-radiotherapy in sub-Saharan Africa. The findings reveal a high prevalence of sexual dysfunction across multiple domains, with cervical cancer patients experiencing diminished desire, poor arousal, lubrication difficulties, and impaired orgasmic function, leading to low relationship satisfaction and avoidance of sexual activity. Similarly, prostate cancer patients demonstrated severe erectile dysfunction, low sexual desire, and dissatisfaction with intercourse and overall sexual experience. Comparisons with previous studies confirm the consistency of these findings across similar cultural settings, reinforcing the need for multidisciplinary interventions that integrate medical, psychological, and cultural considerations. Addressing these challenges is crucial for improving the overall quality of life of cancer survivors in the region.

## Recommendations

Clinicians should incorporate sexual health assessments into routine survivorship care for patients with prostate and cervical cancers, and offer tailored interventions. Partner involvement and culturally sensitive methodologies are essential for a holistic understanding of the impact of cervical cancer on sexual health and relationships. By addressing these gaps, healthcare providers can develop comprehensive survivorship care models that improve the well-being of cervical and prostate cancer survivors. Future research should focus on larger, multicenter, population-based studies to enhance generalizability and include longitudinal designs to capture changes over time. Additionally, future research should control for potential confounders such as age, disease duration, and treatment modality to provide a more nuanced understanding of the factors influencing sexual dysfunction. Qualitative studies exploring patients’ experiences could provide deeper insights into the psychosocial aspects of sexual dysfunction and guide interventions tailored to patient needs.
